# Micronutrient Profiles and Anxiety in Adolescents with Non-Structural Palpitations: A Case–Control Study

**DOI:** 10.3390/nu18050717

**Published:** 2026-02-24

**Authors:** Damla Erden, Ajda Mutlu Mıhçıoğlu, Omer Okuyan, Merve Tarı, Hafize Uzun

**Affiliations:** 1 Department of Pediatric Cardiology, Faculty of Medicine, Istanbul Atlas University, Istanbul 34408, Turkey; 2 Department of Pediatric Cardiology, Bakırköy Dr. Sadi Konuk Training and Research Hospital, Istanbul 34147, Turkey; ajdamutlu@yahoo.com; 3 Department of Pediatrics, Faculty of Medicine, Istanbul Atlas University, Istanbul 34408, Turkey; dmemhs@gmail.com; 4 Department of Clinical Psychology, Istanbul Atlas University Hospital, Istanbul 34203, Turkey; pskmervekoca@gmail.com; 5 Department of Medical Biochemistry, Faculty of Medicine, Istanbul Atlas University, Istanbul 34408, Turkey; huzun59@hotmail.com

**Keywords:** adolescent palpitations, micronutrients, magnesium, selenium, ferritin, anxiety, autonomic dysfunction

## Abstract

**Background**: Palpitations are common in adolescents and often occur without structural heart disease. Although anxiety and autonomic dysregulation have been implicated, the role of micronutrient status remains unclear. This study aimed to investigate the association between palpitations, micronutrient levels, and anxiety in adolescents and to evaluate the independent associations between selected micronutrients and palpitations using multivariable regression models. **Methods**: This case–control study included 52 adolescents with palpitations and 52 frequency-matched healthy controls. Structural heart disease was excluded by electrocardiography, echocardiography, and 24-h Holter monitoring. **Results**: Adolescents with palpitations had significantly lower serum magnesium, selenium, and ferritin levels and higher anxiety scores than controls, despite most values remaining within reference ranges. In age- and sex-adjusted analyses, lower magnesium and selenium levels were independently associated with palpitations. **Conclusions**: Subclinical differences in micronutrient status, particularly magnesium and selenium, together with increased anxiety, may contribute to non-structural palpitations in adolescents. These findings support a more integrative evaluation that includes micronutrient and psychological assessment alongside standard cardiac investigations.

## 1. Introduction

Palpitations are among the most common reasons for medical consultation in adolescents and frequently occur in the absence of an underlying structural cardiac pathology. The persistence of symptoms despite normal findings on electrocardiography (ECG), transthoracic echocardiography (TTE), and 24-h ambulatory electrocardiographic monitoring suggests that the sensation of palpitations may not be solely cardiac in origin, but rather associated with autonomic nervous system dysfunction, stress responses, and anxiety. Increased sympathetic activity and reduced vagal tone have been linked to a predisposition to sinus tachycardia and heightened perception of cardiac sensations, including palpitations [1,2,3].

Micronutrients play a critical role in fundamental biological processes, including cardiac conduction, cellular energy production, neurotransmitter synthesis, and the regulation of oxidative stress. Suboptimal levels or deficiencies in vitamin B12, vitamin D, iron, magnesium, zinc, and selenium may influence autonomic regulation and heart rate variability (HRV) through effects on oxidative balance, neuroendocrine pathways, and metabolic homeostasis. In particular, selenium, due to its role in antioxidant defense mechanisms and thyroid hormone metabolism, has emerged as a biologically relevant factor in cardiovascular and autonomic function [4,5,6].

Although associations between micronutrient deficiencies and autonomic dysfunction have been demonstrated in adult populations, studies investigating the relationship between micronutrient levels and palpitations in adolescents with non-structural palpitations remain limited. Adolescence is a period characterized by pronounced hormonal variability and a high prevalence of micronutrient deficiencies, making this population particularly relevant for investigation [7,8,9].

Micronutrients such as iron, magnesium, and selenium are involved in autonomic nervous system regulation, myocardial electrophysiology, and neuropsychiatric function. Deficiencies in these micronutrients have been associated with increased autonomic excitability, somatic symptom amplification, and anxiety-related cardiovascular complaints. Adolescence represents a period of accelerated growth and increased physiological demand, during which subtle micronutrient imbalances and psychological vulnerability may together contribute to the development of non-structural palpitations [3,7].

The present study aimed to evaluate the relationship between palpitations, micronutrient levels, and anxiety in adolescents without structural heart disease. We hypothesized that adolescents presenting with non-structural palpitations would exhibit lower serum micronutrient levels compared with age- and sex-matched controls, together with a notable anxiety burden within the patient group. A central premise of this study is that differences in mean micronutrient levels between patient and control groups may be clinically meaningful even when values fall within established reference ranges. This approach seeks to provide a more holistic assessment of often-overlooked biochemical contributors in adolescents presenting with palpitations. To the best of our knowledge, there are no studies in existing literature that have specifically investigated this association in adolescents without structural heart disease.

## 2. Materials and Methods

### 2.1. Subjects

This prospective case–control study protocol was approved by the Local Clinical Research Ethics Committee of Istanbul Atlas University (approval number: 08/64; approval date: 29 September 2025). All procedures were performed in accordance with the principles of the Declaration of Helsinki, and written informed consent was obtained from all participants and/or their legal guardians prior to enrollment.

A total of 104 adolescents were included in the study, comprising 52 patients with non-structural palpitations and 52 healthy controls. Age and sex comparability between groups was ensured at the group level (frequency matching) by recruiting participants within the same age range (13–18 years) and achieving similar age and sex distributions across groups.

### 2.2. Participants

For the purposes of this study, “non-structural palpitations” was operationally defined as recurrent subjective palpitation complaints in adolescents aged 13–18 years, in the absence of structural heart disease on echocardiography and without clinically significant arrhythmias on 24-h ambulatory ECG monitoring. Sinus tachycardia episodes were considered physiological rhythm responses rather than pathological arrhythmias.

### 2.3. Inclusion and Exclusion Criteria

Inclusion criteria for the patient group were adolescents aged 13–18 years presenting with palpitations, normal findings on ECG, transthoracic echocardiography (TTE), and 24-h ambulatory electrocardiographic monitoring, absence of pathological arrhythmias other than sinus tachycardia, and provision of written informed consent.

Exclusion criteria (applied to both groups) included the presence of structural heart disease. Trivial or mild valvular regurgitation without associated structural abnormalities or hemodynamic consequence was considered a physiological finding and was not classified as structural heart disease. Detection of supraventricular tachycardia, premature ventricular complexes, or ventricular ectopy on 24-h ambulatory electrocardiographic monitoring; known systemic disease or chronic medication use; history of congenital infection, neurological disease, or intellectual disability; previous diagnosis of anxiety or depressive disorders and/or ongoing psychiatric treatment; and refusal to provide informed consent.

### 2.4. Patient Group

Participants in the patient group were consecutively recruited from the pediatric cardiology outpatient clinic among adolescents aged 13–18 years presenting with palpitations and no abnormal findings on physical examination. Patients with documented arrhythmia on 24-h ambulatory electrocardiographic monitoring, structural cardiac abnormalities on transthoracic echocardiography, metabolic or chronic systemic disease, or regular medication use were excluded.

### 2.5. Control Group

The control group consisted of age- and sex-matched healthy adolescents recruited from the general pediatrics outpatient clinic during routine health check-up visits. Control participants had no history of palpitations, no signs of acute infection at presentation, normal physical examination findings, and no known cardiac, metabolic, or chronic systemic disease. None of the control subjects underwent evaluation for anemia, menstrual abnormalities, fatigue, or suspected micronutrient deficiency, and none were receiving nutritional supplementation at the time of enrollment.

Anthropometric measurements were evaluated using age- and sex-specific percentile references according to World Health Organization (WHO) growth standards.

### 2.6. Sample Collection and Biochemical Parameters Assessment

Venous blood samples were obtained from all participants after an overnight fasting period. Serum samples were separated by centrifugation at 3000 rpm for 10 min and stored at −80 °C until analysis. Biochemical parameters were measured in the central laboratory of a tertiary care hospital using automated clinical chemistry analyzers according to standard routine laboratory procedures.

### 2.7. Statistical Analysis

Statistical analyses were performed using SPSS (version 26.0; IBM Corp., Armonk, NY, USA) and Python (version 3.9.7; Python Software Foundation, Wilmington, DE, USA). Continuous variables were assessed for normality using visual inspection and the Shapiro–Wilk test. Normally distributed variables are reported as mean ± standard deviation and were compared using independent-samples *t*-tests. Non-parametric Mann–Whitney U tests were conducted as sensitivity analyses where appropriate.

To account for multiple comparisons, false discovery rate (FDR) adjustment was applied.

Multivariable logistic regression analysis was performed to evaluate independent associations between selected micronutrients and palpitations after adjustment for age and sex. To improve clinical interpretability and avoid unit-scaling artifacts, serum magnesium was rescaled to represent 0.1 mg/dL increments in regression models. Odds ratios are reported per 0.1 mg/dL increase.

## 3. Results

A total of 104 adolescents were included in the study, comprising 52 patients presenting with palpitations and 52 age- and sex-matched healthy controls. Baseline demographic and clinical characteristics of the study groups are summarized in Table 1. There were no significant differences between the groups with respect to age, body mass index, or anthropometric percentiles (height, weight, and BMI percentiles), indicating adequate comparability.

Biochemical comparisons between adolescents with palpitations and healthy controls are summarized in Table 2. Continuous variables are presented as mean ± standard deviation. Group differences were evaluated using independent-samples *t*-tests, and false discovery rate (FDR) correction was applied to account for multiple comparisons. Non-parametric sensitivity analyses yielded directionally consistent findings. Adolescents with palpitations exhibited significantly lower serum magnesium and selenium concentrations compared with controls. Magnesium levels were 1.97 ± 0.13 mg/dL in patients versus 2.09 ± 0.17 mg/dL in controls (*p* < 0.001). Selenium levels were also significantly reduced in the patient group. These differences remained statistically significant after FDR adjustment. Ferritin concentrations were lower in patients compared with controls and demonstrated borderline statistical significance. Zinc and folate levels were also lower at the group level; however, these differences did not retain statistical significance after correction for multiple testing. No significant differences were observed in vitamin D levels between groups. Likewise, lipid parameters—including total cholesterol, HDL-C, LDL-C, and triglycerides—did not differ significantly between patients and controls. Inflammatory markers, thyroid function tests, and fasting glucose values were also comparable between groups. Importantly, although most micronutrient concentrations remained within laboratory reference ranges, group-level shifts in magnesium and selenium were statistically and potentially biologically meaningful.

Age- and sex-adjusted multivariable logistic regression results are presented in Table 3. To improve interpretability and avoid scaling artifacts, serum magnesium was rescaled to represent 0.1 mg/dL increments. In the adjusted model, higher magnesium levels were independently associated with lower odds of palpitations (adjusted OR 0.54 per 0.1 mg/dL increase; 95% CI 0.43–0.79; *p* = 0.001). Selenium also remained independently associated with palpitations, whereas ferritin demonstrated a borderline association. Age and sex were not significantly associated with the outcome. These findings indicate that the association between magnesium and palpitations is robust and not attributable to confounding by age or sex.

## 4. Discussion

The most important finding of this study is that adolescents presenting with palpitations in the absence of structural heart disease exhibit a distinct biochemical profile and a notable anxiety burden within the patient group. Specifically, serum magnesium, selenium, and ferritin levels were significantly lower in the patient group, despite largely remaining within conventional laboratory reference ranges, highlighting the potential clinical relevance of subclinical micronutrient differences. In parallel, anxiety severity was prominent within the patient group, with moderate-to-severe anxiety predominating among adolescents presenting with palpitations. Multivariable and confounder-adjusted analyses demonstrated that lower magnesium and selenium levels were independently associated with the presence of palpitations. Collectively, these findings suggest that non-structural palpitations in adolescents are not solely benign or idiopathic symptoms but may reflect a potential interaction between subtle biochemical insufficiencies and heightened autonomic–psychological vulnerability.

Vitamin D deficiency is prevalent worldwide, particularly among children and adolescents [10]. Previous studies have highlighted the widespread prevalence of vitamin D deficiency and its potential associations with cardiometabolic and systemic health outcomes in pediatric populations [11]. In contrast to these population-based observations, serum vitamin D levels did not differ significantly between adolescents with palpitations and healthy controls, nor were there significant group differences in total cholesterol, HDL-C, LDL-C, or triglyceride levels. These results suggest that, in our study population, palpitations are unlikely to be mediated through vitamin D–related lipid alterations and may instead involve non-metabolic mechanisms such as autonomic dysregulation or altered symptom perception. Although the mechanisms underlying this association are not fully understood, vitamin D is known to influence lipid metabolism through its effects on parathyroid hormone and calcium homeostasis, which in turn affect lipid synthesis and transport [12].

Our findings are consistent with the review by Lopresti [9], which demonstrated that several micronutrients—including magnesium, selenium, iron, and B vitamins—are closely associated with heart rate variability (HRV), a key marker of autonomic nervous system regulation. Importantly, even suboptimal micronutrient status within laboratory reference ranges has been shown to adversely affect sympathovagal balance and cardiac autonomic control [9,10,11,12,13]. In parallel, Parker et al. [14] reported that altered HRV in children and adolescents is associated with autonomic dysregulation and increased emotional vulnerability, potentially enhancing sensitivity to internal bodily signals. Within this framework, the observed associations between lower magnesium and selenium levels and palpitations in our adolescent cohort are in line with the hypothesis that subtle micronutrient insufficiencies may contribute to autonomic imbalance and heightened cardiac symptom perception, even in the absence of structural heart disease.

Intervention studies examining the relationship between magnesium supplementation and HRV have yielded heterogeneous results. While no significant changes in HRV parameters were observed in studies involving acute intravenous administration or short-term oral supplementation [15,16,17], other evidence suggests a potential beneficial effect. In patients with cardiovascular disease, oral magnesium supplementation for five weeks was associated with improvements in HRV complexity when assessed using nonlinear methods, which may be more sensitive to autonomic alterations than conventional linear metrics [18,19]. Similarly, improvements in HRV parameters have been reported in healthy adults following longer-term magnesium supplementation [20]. Taken together, these findings indicate that the impact of magnesium on autonomic regulation may depend on baseline health status, supplementation duration, and methodological approaches. In this context, the association observed in our study between lower magnesium levels and palpitations supports a possible link between subclinical magnesium insufficiency and altered autonomic balance, potentially influencing cardiac symptom perception.

Micronutrient inadequacies are widely recognized as an important public health issue in children and adolescents aged 3–17 years, with potential negative consequences for growth, immune function, and cognitive development. Nationally representative data from China have identified priority concerns, including vitamin D deficiency in adolescents, iron deficiency among adolescent girls, and suboptimal selenium status in rural populations [21]. Consistent with these observations, adolescents with non-structural palpitations in our study exhibited significantly lower serum selenium levels compared with healthy controls, even when values remained within reference ranges. Fayet-Moore et al. [22] similarly reported that suboptimal selenium status is common among young adults, highlighting selenium as a potentially overlooked micronutrient during periods of increased physiological demand. Together, these findings suggest that relative selenium insufficiency may be associated with clinically relevant alterations in autonomic regulation and symptom manifestation beyond population-based deficiency thresholds.

There is currently no clear consensus regarding routine micronutrient assessment during pediatric follow-up, as reflected in both U.S. and European clinical guidelines. Although micronutrients are essential for numerous physiological processes, clinical manifestations of insufficiency often emerge only at later stages, limiting early detection. In this context, despite most micronutrient values remaining within laboratory reference ranges, adolescents with palpitations exhibited significantly lower group-level concentrations of selenium, magnesium, ferritin, folate, vitamin B12, and zinc compared with healthy controls [23,24,25,26,27]. This observation supports the concept that micronutrient-related physiological effects occur along a continuum rather than at fixed deficiency thresholds, particularly in systems sensitive to autonomic regulation and neuromuscular excitability. Moreover, population-level shifts in micronutrient distribution—even within reference limits—have been shown to influence oxidative balance, mitochondrial function, and sympathovagal tone [28]. Dietary influences on HRV also appear to be bidirectional, with certain nutrients supporting autonomic regulation, whereas unfavorable dietary components may impair HRV and resting heart rate [29].

Iron and zinc are highly bioavailable from animal-derived foods and frequently co-occur in the diet, which may contribute to concurrent marginal deficiencies. Along with copper and selenium, these trace elements are involved in multiple metabolic pathways. Zinc plays a critical role in growth, immune function, reproductive health, and neurobehavioral development, and serum or plasma zinc concentration remains the primary recommended biomarker for population-level assessment [30,31,32,33]. Disturbances in zinc homeostasis have been linked to oxidative stress, endothelial dysfunction, and adverse changes in antioxidant capacity, despite inconsistent findings regarding atherosclerosis [34,35,36]. In line with these biological roles, adolescents with non-structural palpitations in our study exhibited lower serum zinc levels compared with healthy controls. The concurrent reductions in zinc, magnesium, and selenium may indicate that multiple trace element insufficiencies coexist, potentially influencing autonomic regulation and cardiac symptom perception during adolescence.

Vitamins B12 and folate are essential for DNA synthesis, hemoglobin production, and homocysteine metabolism, with deficiencies linked to megaloblastic anemia and neurological or metabolic disturbances [7,8,9,10,11,12,13,14,15,16,17,18,19,20,21,22,23,24,25,26,27,28,29,30,31,32,33,34,35,36,37]. Notably, Sucharita et al. [37] reported that even subclinical vitamin B12 deficiency was associated with altered HRV across the life course, highlighting a potential mechanistic link between micronutrient status and autonomic regulation. Consistent with these findings, adolescents with palpitations in our study exhibited lower group-level concentrations of vitamin B12 and folate compared with healthy controls, despite values remaining within reference ranges. These results are consistent with the concept that subtle micronutrient insufficiencies during critical developmental periods may contribute to autonomic vulnerability and heightened cardiac symptom perception.

### 4.1. Strengths and Limitations of the Study

This study has several notable strengths. First, it focuses on a well-defined adolescent population with palpitations in whom structural heart disease was rigorously excluded using electrocardiography, echocardiography, and Holter monitoring. This strict exclusion of organic cardiac pathology allowed for a more precise evaluation of non-structural contributors to palpitations. Second, the simultaneous assessment of multiple micronutrients together with anxiety severity provides a comprehensive and integrative perspective that extends beyond conventional cardiac evaluation. The use of multivariable logistic regression with age- and sex-adjustment, together with sensitivity analyses, strengthens the robustness of the findings and reduces the likelihood of confounding. Third, the demonstration of clinically meaningful differences in micronutrient levels despite values remaining within laboratory reference ranges represents an important conceptual contribution, highlighting the potential limitations of relying solely on dichotomous “normal/abnormal” thresholds in clinical practice.

Several limitations should be acknowledged. The case–control design precludes causal inference and does not establish temporality between micronutrient status, anxiety, and palpitations. In addition, detailed quantification of palpitation frequency, duration, triggers, and real-time symptom–Holter correlation was not systematically documented, which may introduce heterogeneity in the clinical phenotype. Selection bias is possible because participants were recruited from a single center, which may limit generalizability.

Micronutrients were measured at a single time point; dietary intake, supplementation, seasonal variability (particularly for vitamin D), and intra-individual fluctuations were not assessed, introducing potential measurement error and exposure misclassification. Although models were adjusted for age and sex, residual confounding from unmeasured factors (e.g., socioeconomic status, dietary patterns, pubertal stage, physical activity, inflammation, stress, and sleep quality) cannot be excluded. Because anxiety was not assessed in the control group, it could not be included as an adjustment variable in the multivariable case–control models, and its potential role as an interacting or mediating factor cannot be fully excluded.

The initially extreme OR for magnesium reflected unit scaling due to its narrow biological range; therefore, rescaled estimates (per 0.1 mg/dL) are reported for interpretability. Finally, autonomic dysfunction was inferred indirectly rather than measured directly (e.g., HRV), and some degree of statistical instability or model heterogeneity may be present, particularly in light of wide confidence intervals in certain regression estimates. Therefore, findings should be interpreted cautiously and validated in larger, multicenter cohorts.

### 4.2. Clinical Implications

These findings suggest that palpitations in adolescents without structural heart disease may warrant a broader, integrative clinical assessment even when routine cardiac investigations are normal. Although causality cannot be inferred, the observed group-level differences in micronutrient status—particularly magnesium and selenium—together with a higher anxiety burden indicate that nutritional and psychosocial factors may contribute to symptom perception and persistence. In clinical practice, a comprehensive clinical evaluation that includes consideration of nutritional status and psychological well-being may help contextualize symptoms in adolescents with recurrent palpitations after exclusion of structural or arrhythmic pathology, potentially supporting more individualized management and reducing repetitive, low-yield diagnostic testing. Further prospective studies are required to determine whether targeted correction of micronutrient insufficiency and/or anxiety-focused interventions improve clinical outcomes.

### 4.3. Future Perspectives

Future studies should aim to validate these findings in larger, multicenter cohorts to enhance generalizability and statistical power. Longitudinal designs are needed to clarify causal relationships and to determine whether correction of subclinical micronutrient deficiencies or targeted anxiety management leads to improvement in palpitations. Incorporation of direct autonomic function measures, such as HRV or baroreflex sensitivity, may further elucidate the mechanistic pathways linking micronutrient status, anxiety, and cardiac symptom perception. Interventional trials evaluating magnesium or selenium supplementation, either alone or in combination with psychological interventions, may help clarify their potential role in personalized and integrative management strategies for adolescents with non-structural palpitations.

## 5. Conclusions

In conclusion, adolescents with palpitations in the absence of structural heart disease exhibit lower magnesium, selenium, and ferritin levels, even when biochemical values largely remain within conventional reference ranges, together with a substantial anxiety burden observed within the patient group. Multivariable and confounder-adjusted analyses identified magnesium and selenium as independently associated factors for palpitations. These findings indicate that non-structural palpitations in adolescents may reflect an interaction between subtle micronutrient insufficiencies and psychological–autonomic vulnerability rather than a purely benign or idiopathic phenomenon. Accordingly, a more integrative clinical approach that incorporates micronutrient assessment alongside psychological evaluation may be warranted in the evaluation and management of this population.

## Figures and Tables

**Table 1:** Demographic and Clinical Characteristics of Study Groups.

Parameter	Patients (*n*= 52)	Controls (*n* = 52)
Age (years)	14.48 ± 1.78	14.63 ± 1.73
Sex	Female: 34 (65.4%); Male: 18 (34.6%)	Female: 27 (51.9%); Male: 25 (48.1%)
Body Mass Index (kg/m^2^)	22.54 ± 4.35	23.15 ± 2.85
BMI percentile (WHO)	70 (45–92.5)	60 (45–80)
Height percentile (WHO)	55 (25–90)	55 (50–75)
Weight percentile (WHO)	67.5 (45–97.5)	65 (50–85)
QTc interval (ms)	384.42 ± 21.09	-
Mean heart rate (Holter, bpm)	87.44 ± 11.19	-
Maximum heart rate (Holter, bpm)	157.60 ± 10.34	-
ECG rhythm	Normal sinus rhythm: 52 (100.0%)	-
Sinus tachycardia episodes (Yes/No)	Yes: 50 (96.2%); No: 2 (3.8%)	-
Echocardiography findings	Normal findings (no structural pathology): 42 (80.8%); Mild mitral regurgitation: 8 (15.4%); Mild aortic regurgitation: 2 (3.8%)	-
BAI score	20.15 ± 12.74	-
BAI category	Normal: 6 (11.5%); Mild anxiety: 14 (26.9%); Moderate anxiety: 21 (40.3%); Severe anxiety: 11 (21.1%)	-

Data are presented as mean ± standard deviation for continuous variables, median (interquartile range) for percentile-based anthropometric variables, and number (percentage) for categorical variables. Anthropometric percentiles were calculated using age- and sex-specific World Health Organization (WHO) growth standards. Clinical, electrocardiographic, Holter, echocardiographic, and anxiety-related parameters were assessed only in the patient group.

**Table 2:** Biochemical Parameters in Patients and Controls.

Parameter	Patients (Mean ± SD)	Controls (Mean ± SD)	Test	*p* Value	FDR-Adjusted *p*	Reference Range
Hemoglobin (Hb)	13.44 ± 1.55	13.35 ± 1.59	*t*-test	0.751	0.890	12.0–16.0 g/dL
Hematocrit (Hct)	40.32 ± 4.10	40.63 ± 6.64	*t*-test	0.774	0.890	36–46%
RBC	4.95 ± 0.43	5.06 ± 1.19	*t*-test	0.529	0.890	3.90–5.40 × 10^6^/µL
WBC	6.90 ± 1.46	7.62 ± 1.80	*t*-test	**0.026**	0.214	4.27–11.40 × 10^3^/µL
PLT	280.23 ± 60.71	295.75 ± 60.14	*t*-test	0.193	0.572	132–395 × 10^3^/µL
MCV	81.02 ± 5.91	80.75 ± 5.56	*t*-test	0.811	0.890	79.8–96.7 fL
MCH	26.86 ± 2.64	26.55 ± 2.21	*t*-test	0.526	0.638	25.7–31.4 pg
MCHC	33.18 ± 1.21	33.04 ± 1.11	*t*-test	0.550	0.833	31.8–36.4 g/dL
Vitamin B12	386.38 ± 133.33	438.85 ± 150.91	*t*-test	0.063	0.228	182–820 ng/L
Vitamin D	23.56 ± 11.13	27.29 ± 13.16	*t*-test	0.122	0.416	20–80 ng/mL
Folic Acid	5.65 ± 2.76	7.46 ± 4.02	*t*-test	**0.009**	0.136	2.8–17.1 ng/mL
Iron (Fe)	81.41 ± 48.17	84.02 ± 41.10	*t*-test	0.767	0.833	40–160 µg/dL
Ferritin	34.22 ± 30.62	43.80 ± 24.17	*t*-test	0.080	**0.025**	7–84 µg/L
Magnesium (Mg)	1.97 ± 0.13	2.09 ± 0.17	*t*-test	**<0.001**	**0.025**	1.9–2.5 mg/dL
Calcium (Ca)	9.99 ± 0.39	10.03 ± 0.34	*t*-test	0.568	0.857	8.8–10.6 mg/dL
Potassium (K)	4.40 ± 0.32	4.34 ± 0.30	*t*-test	0.289	0.572	3.5–5.1 mmol/L
Sodium (Na)	138.08 ± 1.80	137.98 ± 1.84	*t*-test	0.788	0.890	132–145 mmol/L
Selenium	64.81 ± 11.71	72.06 ± 12.60	*t*-test	**0.003**	0.092	55–134 µg/L
Zinc	98.42 ± 16.82	105.04 ± 14.37	*t*-test	**0.033**	0.170	70–114 µg/dL
Copper	108.05 ± 21.29	108.88 ± 21.16	*t*-test	0.841	0.890	70–150 µg/dL
Total Protein	76.69 ± 4.56	76.81 ± 4.95	*t*-test	0.897	0.963	6.3–8.0 g/L
Albumin	46.71 ± 7.84	47.55 ± 6.15	*t*-test	0.546	0.890	38–54 g/L
Total Cholesterol	161.83 ± 31.20	162.19 ± 30.62	*t*-test	0.952	0.995	<150 mg/dL
HDL	54.29 ± 12.95	57.15 ± 13.36	*t*-test	0.271	0.572	35–60 mg/dL
LDL	84.11 ± 25.22	85.22 ± 24.26	*t*-test	0.819	0.890	<100 mg/dL
Triglycerides	109.98 ± 53.60	114.24 ± 52.02	*t*-test	0.682	0.833	0–150 mg/dL
AST	18.74 ± 5.12	21.70 ± 8.63	*t*-test	**0.037**	0.422	0–31 U/L
ALT	15.39 ± 7.31	16.02 ± 6.07	*t*-test	0.630	0.517	0–34 U/L
CRP	1.48 ± 1.29	1.51 ± 1.27	*t*-test	0.897	0.880	<5 mg/L
TSH	2.41 ± 1.10	2.24 ± 0.97	*t*-test	0.403	0.639	0.55–4.78 µIU/mL
Free T4	2.70 ± 3.91	1.97 ± 2.89	*t*-test	0.278	0.422	0.79–2.8 ng/dL
Fasting blood glucose	94.77 ± 9.59	92.50 ± 10.83	*t*-test	0.261	0.890	70–100 mg/dL

Data are presented as mean ± SD. Group comparisons used independent-samples *t*-tests. For transparency, FDR-adjusted *p* values from non-parametric sensitivity analyses (Mann–Whitney U) are also provided, addressing multiple testing and distributional robustness. Statistically significant *p* values (*p* < 0.05) are shown in bold.

**Table 3:** Confounder-Adjusted Multivariable Logistic Regression Analysis.

Variable	Adjusted OR	95% CI (Lower)	95% CI (Upper)	*p* Value
Magnesium (per 0.1 mg/dL increase)	0.540	0.430	0.790	**0.001**
Selenium	0.961	0.925	0.997	**0.035**
Ferritin	0.985	0.968	1.001	0.073
Age	0.949	0.740	1.217	0.682
Female	1.505	0.596	3.798	0.387

Age was entered as a continuous variable and sex was coded as female vs. male. Statistically significant *p* values (*p* < 0.05) are shown in bold.

## Data Availability

The data presented in this study are available on request from the corresponding author. The data are not publicly available due to privacy and ethical restrictions.
